# Observations on the Use of Professor Keil's Magneto-Electric Apparatus in Various Diseases, Especially in Chronic Neuroses, Rheumatic, and Gouty Affections

**Published:** 1843-07

**Authors:** 


ART. V.—*Beobachtungen über den Nützen und Gebrauch des Keilschen Magnet-Electrischen Rotations apparats in Krankheiten, &c.* Von J. E. WETZLER.—*Leipzig,* 1842. 8vo, pp. 182.

*Observations on the Use of Professor Keil's Magneto-Electric Apparatus, in various diseases, especially in Chronic Neuroses, Rheumatic and Gouty Affections.* By J. E. WETZLER.—*Leipzig,* 1842.

THE book before us cannot be considered as a scientific treatise on the subject of which it professes to treat, as it is but a record of cases; of cures performed, partially accomplished, or attempted; with a brief notice at the end, of the apparatus employed. The history of these cases, ninety-five in number, occupies 152 pages of this little volume. They are detailed with a minuteness and fidelity peculiar to the German character, but throughout the whole there runs so strong a predilection for the favorite remedy, that we are naturally rendered cautious in crediting its marvellous powers. The apparatus of Professor Keil is minutely described, and it appears in many points to possess advantages over the first machine invented by Professor Faraday in 1833. The remarkable properties of electro-magnetism have been admirably illustrated by Professor Oersted of Copenhagen, while the weaker agency of the magneto-electric rotation apparatus of Faraday, has been but little attended to by medical men in England. To the speculative Germans, however, the mystic powers of the magnet combined with those of electricity presented too inviting a field for investigation, to be neglected; and we accordingly find that Dr. Wetzler is but one of several physicians who have availed themselves of these properties for the cure of disease. The apparatus of Professor Kiel has been employed in the following disorders:

*In affections of the organs of sight.* Three cases are given, two of which are far from satisfactory; the third was a case of rheumatic amaurosis of the right eye, in consequence of a sabre cut, which had injured the organs of sight. After three or four applications of the remedy, the sight, which was previously almost entirely lost, improved, and after twelve sittings the patient was enabled to distinguish the window panes at the distance of fifteen feet, but a complete cure was not effected.

*Organs of hearing.* Of nine cases of deafness, most of which appeared to be connected with abdominal plethora, only two could be reported completely cured, the other patients, though relieved for a time, either lost faith in the prolonged administration of the remedy, or were obliged to leave the baths before a cure could be effected. Such at least appears to be Dr. Wetzler's conviction, but we should not forget that most of his patients whose cases are recorded in this work employed at the same time regularly the mineral waters of the baths, at which he was exercising his magic skill.

*Neuralgia.* The cases of neuralgia are related at great length, and four or five out of twenty appear really to have been completely cured by the magneto-electric apparatus. But was this a greater number than those who are relieved of severe nervous pains by galvanism, or by other well known remedies?

Dr. Wetzler is free from one great fault of writers of his description; he does not hesitate to record a failure in his practice; and indeed, on analysing carefully the ninety cases he has given to the world, we find that want of success attended not a few. In muscular *rheumatism* his practice was attended with considerable success, but in such cases we find that he only relates his experience of the *immediate* relief his patients obtained from his apparatus; for the majority after returning to their homes, forgot or neglected to transmit to him any account of their future history.

According to our author there are few of the disorders connected with, or arising from impaired digestive powers, which may not be benefited by magneto-electricity; but, if some improvement be not manifested after three or four trials, he does not recommend the continuance of the remedy.

The description of Professor Keil's apparatus is too long for insertion in this brief notice, and this is the less to be regretted, as the author acknowledges that it is greatly exceeded in efficacy and in cheapness, by the machine lately invented by Professor Neef, of Frankfort on the Maine. But of this latter he gives no detailed account, only mentioning his own astonishment at witnessing the powers of the apparatus, and, moreover, that the cost of it (£4 to £6) will place it within the reach of many to whom the more expensive machine of Professor Keil was unattainable.

To conclude, Dr. Wetzler's book contains some useful information, alloyed with much that could only arise from a great deal of credulity: nor can we wonder that the man who was himself restored to health by following the revelations of a somnambulist, should lend a willing ear and ready hand to the little known and mysterious agency of magnetism and electricity.

